# Supplementary material for: MetaRibo-Seq measures translation in microbiomes
Source: Nat Commun. 2020 Jun 29;11:3268. doi: 10.1038/s41467-020-17081-z (PMC7324362; doi:10.1038/s41467-020-17081-z)
Supplement: Supplementary file 10 — Supplementary Data 7 [file 41467_2020_17081_MOESM10_ESM.zip › File2/Confidence_High_Taxonomy/306822_out.krona.html]

Javascript must be enabled to view this page.

members
magnitude
magnitudeUnassigned
count
unassigned
taxon
rank

306822\_out

9

superkingdom
4
2759

kingdom
4751
4

subkingdom
451864
4

phylum
4890
4

147538
4
subphylum

147550
4
class

4
222543
subclass

order
4
5125

4
110618
family

genus
1620264
4

307937
4
species

SRS013687\_contig\_number\_3705SRS023914\_contig\_number\_27650SRS076929\_contig\_number\_26534SRS1041145\_contig\_number\_33991

superkingdom
5
2

1239
2
phylum

class
91061
2

1385
2
order

family
2
186822

genus
44249
2


SRS023914\_contig\_number\_26314SRS051031\_contig\_number\_contig-100\_724.195583
species
1986223
2

phylum
3
976

class
200643
3

order
171549
3

3
1950409

SRS013687\_contig\_number\_4344SRS053356\_contig\_number\_45034SRS1041145\_contig\_number\_35079
species
